# Marine microalgae bioengineered *Schizochytrium sp*. meal hydrolysates inhibits acute inflammation

**DOI:** 10.1038/s41598-018-28064-y

**Published:** 2018-06-29

**Authors:** Xiaoli Wang, Heng Wang, Joseph F. Pierre, Sheng Wang, Huifang Huang, Jun Zhang, Shuangzhen Liang, Qingzhu Zeng, Chenqing Zhang, Meijuan Huang, Chengxu Ruan, Juan Lin, Hao Li

**Affiliations:** 1 Institute of Applied Biotechnology, College of Biological Science and Technology, Fuzhou University, Fuzhou, Fujian 350116 China; 2 Section of Gastroenterology, Hepatology, and Nutrition, Department of Medicine, The University of Chicago, Chicago, IL 60637 USA; 3 Department of Human Genetics, The University of Chicago, Chicago, IL 60637 USA; 4 Central Laboratory, Fujian Medical University Union Hospital, Fuzhou, 350001 China; 5 Fujian Key Laboratory of Marine Enzyme Engineering, Fuzhou University, Fuzhou, Fujian China; 6 Fujian LandhowbioTech. Corp.,Ltd., Fuzhou, Fujian 350108 China

## Abstract

Bioengineered marine microalgae *Schizochytrium* sp. is currently used to produce docosahexaenoic acid (DHA). However, following DHA extraction, the remaining protein-rich materials are not well utilized. In this study, we report that marine microalgae bioengineered *Schizochytrium* sp. hydrolysate (MESH), which exhibits a unique peptide profile as identified by Ultra Performance Liquid Chromatography coupled with Q-TOF mass spectrometry(UPLC/Q-TOF-MS), ameliorated bowel inflammation in mice. In a mouse model of experimentalcolitis induced by dextran sulfate sodium, compared with the control mice, the mice treated with MESH were highly resistant to colitis, as demonstrated by marked reductions in body weight loss, clinical colitis scores, colonic histological damage, and colonic inflammation. Mechanistically, MESH attenuated the induction of pro-inflammatory cytokines and increased the induction of anti-inflammatory cytokines. MESH also promoted the proliferation of colonic crypt stem cells and progenitor cells required for crypt repair. Collectively, these results reveal a previously unrecognized role of MESH as a potential anti-inflammatory treatment for colitis.

## Introduction

The marine microalgae *Schizochytrium* sp. is an important source of docosahexaenoic acid (DHA, C22:6) and has been bioengineered to produce even greater levels of DHA for food manufacturing. Although DHA is critical for optimal human health and function throughout life, humans cannot synthesize DHA, and it must therefore be ingested through diet. To date, research on DHA has focused on how to increase the DHA yield from *Schizochytrium* sp. by improved extraction methods^1,2^ and the study of DHA-mediated processes in the human body, such as nervous system development in infants and the prevention of cardiovascular disorders^3,4^. However, the study of *Schizochytrium* sp. proteins has remained relatively unexplored. Depending on the fermentation method, the total protein content in *Schizochytrium* sp. may reach 9.35 or 42.51% using soybean meal or yeast extract as a nitrogen source, respectively^5^. Moreover, modern bioengineering processes can produce *Schizochytrium* sp. meal with even greater protein levels following DHA extraction, but this product is currently used as feedstuff^6^. Only one study has examined the soluble proteins extracted from *Schizochytrium* sp.^7^, and hydrolysates derived from *Schizochytrium* sp. meal have not been profiled or examined for their potential therapeutic uses.

Despite being necessary for survival, inflammatory processes can cause tissue damage when they are chronically or acutely manifested as immune responses, causing oedema, swelling, pain, and cellular dysfunction. While these biological reactions are necessary to clear infectious and injurious agents through clotting, immune cell diapedesis, and lymphatic drainage, prolonged responses can lead to tissue dysfunction^8^. Specifically, acute inflammatory injuries may induce excessive bleeding, tissue degeneration, or necrosis and may impair long-term homeostasis^9^. Numerous signalling cytokines mediate acute and chronic inflammation. Many inflammatory cytokines are associated with inflammatory bowel diseases (IBD) and can be divided into pro-inflammatory and anti-inflammatory cytokines. These substances play important roles in the pathogenesis of enteritis, as an imbalance between pro- and anti-inflammatory cytokines can result in abnormal immune response. The common pro-inflammatory cytokines are IL-1β, IL-6, IL-17 and TNF-α^10^, while the common anti-inflammatory factors are IL-4, IL-10 and IL-13^11^.

The most broadly used drugs are non-steroidal anti-inflammatory drugs (NSAIDs), which target eicosanoid-producing cyclooxygenases. While effective, some NSAIDS have long-term side effects, including gastrointestinal bleeding, nausea, vomiting and diarrhoea^12^. Drugs used to treat acute and chronic conditions, such as glucocorticoids (GC) and NSAIDS, are indispensable in medical therapies, but the identification of new anti-inflammatory compounds that are complementary to current drug regimens remains desirable^13,14^. In addition, new anti-dermatitis drugs to replace the currently used drugs that have no toxic side effects are required.

In this study, we investigated marine microalgae bioengineered *Schizochytrium* sp.meal hydrolysate (MESH) and its potential anti-inflammatory roles. Although Inflammation occurs in all parts of the body, the primary drug delivery methods used to treat inflammation are oral and injection treatments. Because *Schizochytrium* sp. meal hydrolysates derived from food resources, they will be used by body to primarily depend on the digestive system. Since MESH is not suitable for injection, we chose a DSS-induced animal model to test the efficacy of MESH for its future applications. The selected animal model was a murine inflammatory injury model. Our aim was to explore the mechanism of MESH-related activity, including anti-inflammatory factors, cytokines and cell proliferation. We report, for the first time, the use of bioengineered marine microalgae *Schizochytrium* sp.meal and hydrolysates in a murine injury model, demonstrating the potential for these bioengineered products to be utilized for the treatment of specific inflammatory conditions.

## Results

### Hydrolysate preparation and compositional analysis

The meal produced following DHA extraction from the bioengineered marine microalgae *Schizochytrium* sp. was ground to a powder using a grinder. The meal powder was first digested with pepsin for 1 h at pH 2 followed by a trypsin digestion for 5 h at pH 7, as previously described^15^. The hydrolysate obtained from the two-enzyme process was filtered through membrane filters with a molecular weight cut-off (MWCO) of 1 kDa and centrifuged to separate it into two parts: one part with MWCO more than 1 kDa and another part with MWCO less than 1 kDa in size. The isolated products with MWs of less than 1 kDa were named MESH. A 1 kDa MWCO was chosen based on the theoretical peptides produced from the protein sequences in *Schizochytrium* sp. when cleaved by enzymes. These peptides were then assessed using AQS^16^ to generate alignments with known bioactive peptides. The results showed that the MW of many theoretical bioactive peptides was no more than 1 kDa. Therefore, the peptides filtered with a 1 kDa MWCO filter were used to assess bioactivity.

**Figure 1:**
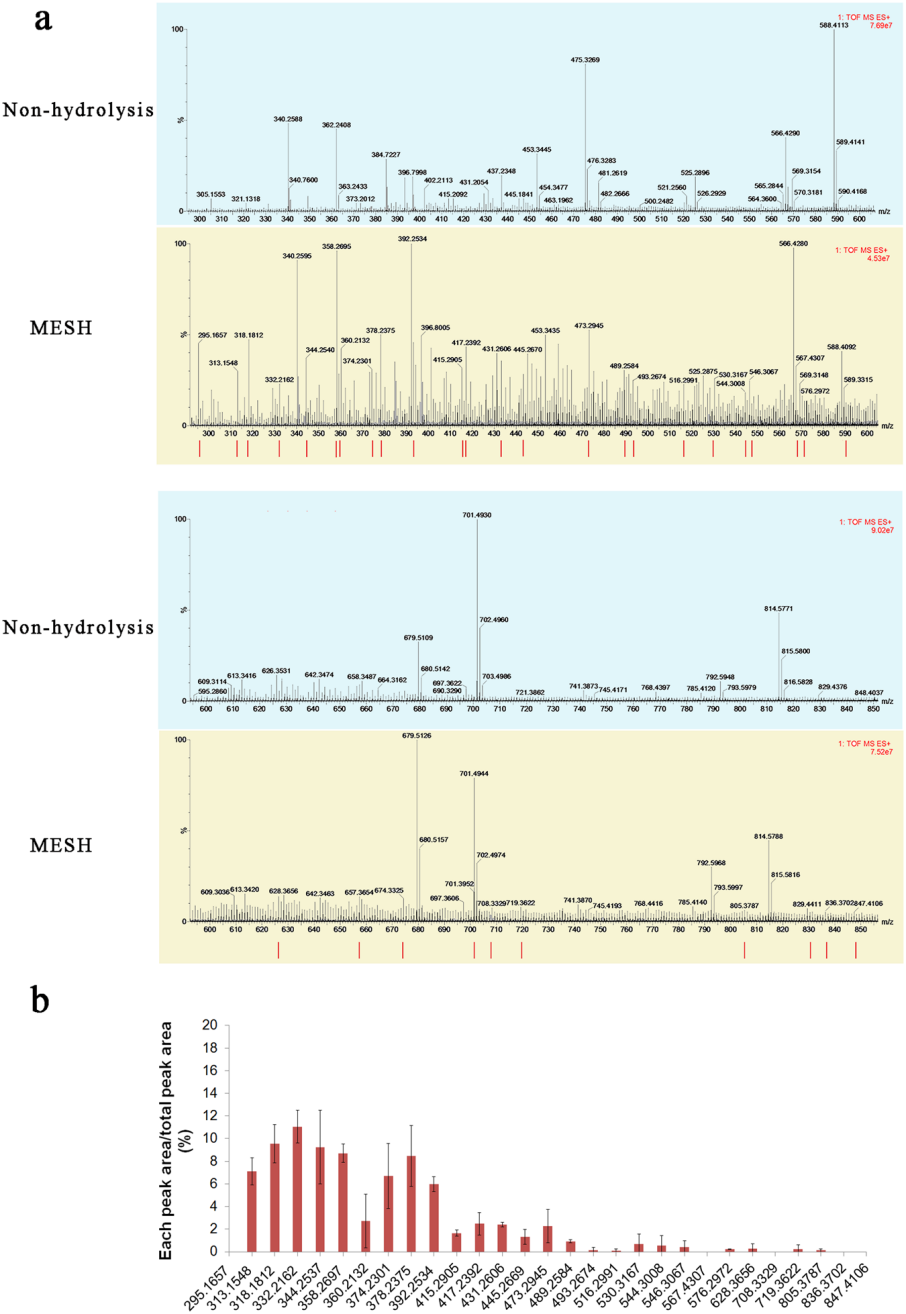
Identification of MESH components. (**a**) UHPLC-Q-TOF/MS mass spectra of MESH and non-hydrolysed meal, exhibiting MWs between 300 and 1000 Da. Red bars indicate the unique MW compounds between the MESH and non-hydrolysed meal resulting from the hydrolysis process. (**b**) The proportion of each peak area over the total areas of the signal for the 34 novel products. Green dots stand for the 2 products with distinct MWs that were observed after hydrolysis but not in the non-hydrolysed meal or the theoretical peptides.

The MESH was freeze dried into a powder and then analysed by UPLC/Q-TOF-MS spectrometry. Unhydrolysed *Schizochytrium* sp. meal, termed ‘non-hydrolysed meal’, was also analysed by UPLC/Q-TOF-MS (Fig. 1a). From comparisons of the resulting spectra, peptides with 34 new MWs were produced by hydrolysis, and different products were identified between the MESH and non-hydrolysed meal analyses. However, there were no other differences in product handling, indicating that these were new breakdown products derived from the starting meal material (Fig. 1b). There were 34 peptides with different MWs distributed between 300 and 850 Da, including those with MWs of 313.15, 318.18, 332.21, 344.25, 358.26, 360.21, 374.23, 378.23, 392.25, 415.29, 417.23, 431.26, 445.26, 473.29, and 489.25 Da. Each peak area/total peak area was between 2 and 12%, showing a high content of peptides. However, each peptide of a given MW may have corresponded to several theoretical peptides (Supplementary Table 2). These different peptide molecules, for which the MW was within 1000 Da, constituted the primary components of the MESH mixture.

### MESH attenuates severe clinical symptoms in a DSS-induced colitis model

**Figure 2:**
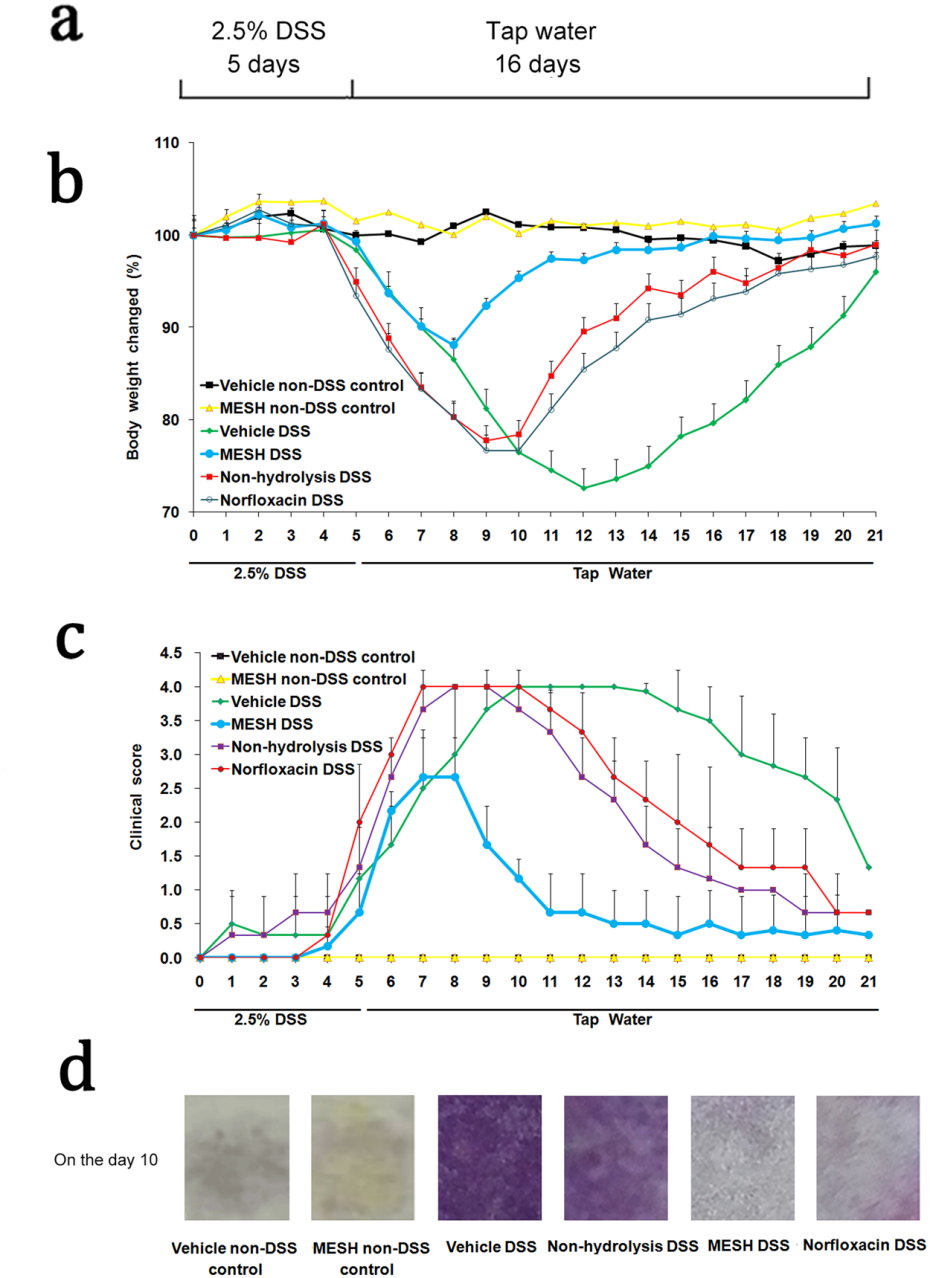
MESH attenuates DSS-induced colitis. (**a**) Schematic illustration of the DSS treatment protocol. (**b**) Body weight changed (percentage of original body weight) over time (days). (**c**) Time course of the DAI in WT mice treated with MESH, non-hydrolysed meal, norfloxacin or vehicle. (**d**) Assay to detect bleeding in the stool when the mice treated with vehicle were showing the most serious disease symptoms on day 10. The purple colour indicates the degree of colonic bleeding. ^*^P < 0.05; ^**^P < 0.01 versus the corresponding vehicle-DSS group, Student’s *t*-test. n = 5–6 mice in each group.

The loss of colonic barrier function as a model of ulcerative colitis was achieved by administration of dextran sulfate sodium (DSS) as previously described^17^. The anti-colitic activity of MESH was then assessed during DSS treatment in wild-type (WT) mice. The mice received one cycle of DSS in drinking water for 5 days, followed by two weeks of recovery with tap water alone (Fig. 2a).

**Figure 3:**
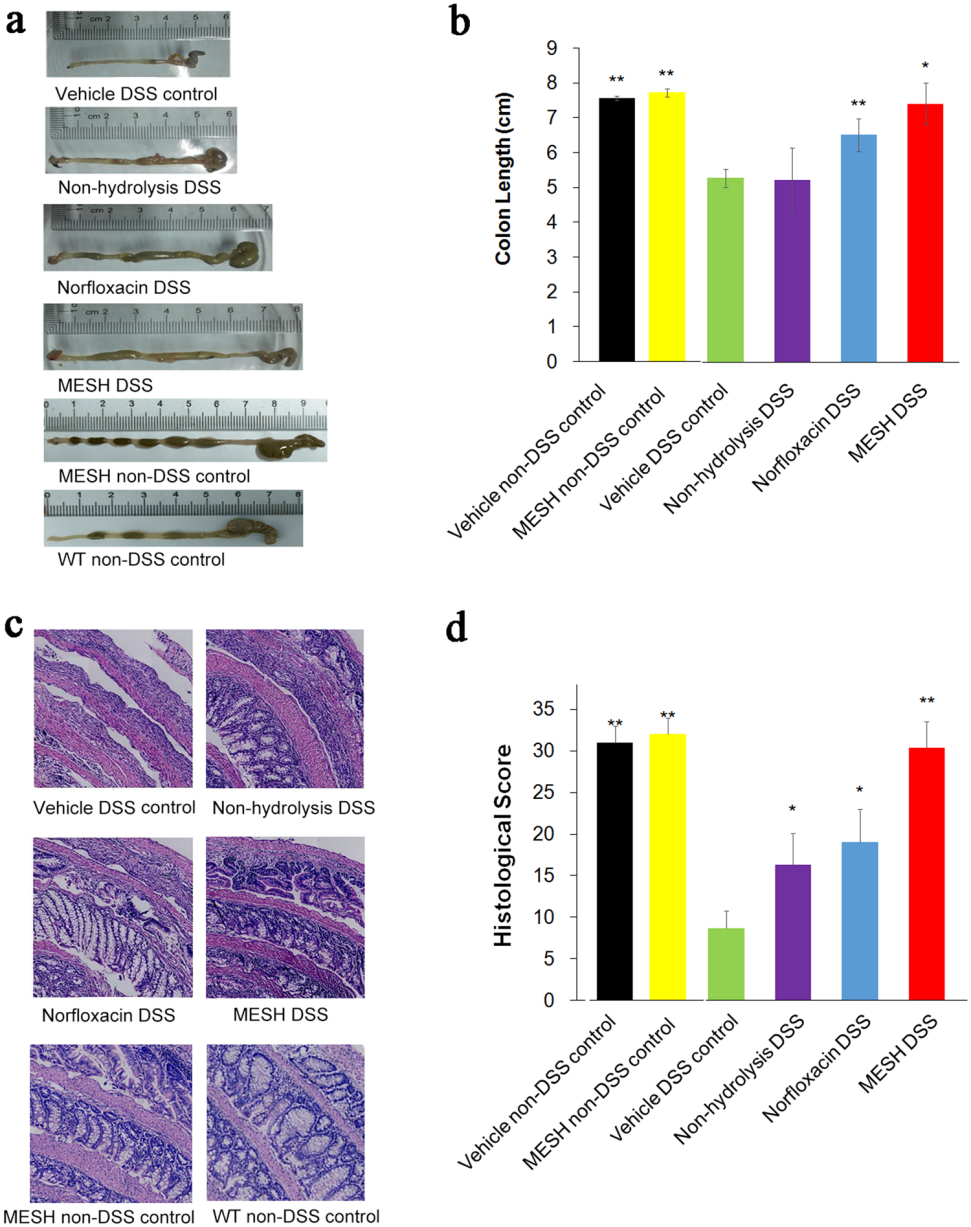
MESH protects against DSS-induced colitis. (**a**) Gross morphology of the large intestine treated with MESH, non-hydrolysed meal, norfloxacin or vehicle on day 10 following DSS treatment. (**b**) The average colon length per treatment group. Vehicle-treated mice displayed the shortest colon lengths, consistent with severe inflammation, while the MESH-treated colons were significantly longer. (**c**) H&E stained colonic sections on day 10. The MESH treatment resulted in the greatest mucosa integrity, while the vehicle group displayed complete loss of crypts and severe colitis. Original magnification, 100×. (**d**) Histological score of four colons from each group on day 10. ^*^P < 0.05; ^**^P < 0.01 versus the corresponding vehicle group, Student’s *t*-test. n = 7 in each group.

The body weights of the mice were measured every day during the experiment. The body weights of the mice in the DSS group began to change on day 4. The body weight loss of the MESH-DSS mouse group was no more than 13%, but that of the vehicle-DSS mouse group was over 25% (Fig. 2b). This result indicated that MESH significantly attenuated body weight loss in the MESH-treated mice, compared to the other DSS group. In contrast, the body weights of the vehicle-non-DSS control and the MESH-non-DSS control groups remained almost unchanged from day 0 to day 21. Clinical scores were assessed daily from the first day of DSS treatment. The mice treated with a vehicle control developed clinical symptoms of colitis with increasing severity starting on day 3, which peaked on day 10 and then recovered slowly until day 21, whereas the MESH-treated mice began recovering on day 8 and demonstrated significantly reduced colitic severity compared with the vehicle controls (Fig. 2c). The mice treated with non-hydrolysed meal showed similar effects to those in the positive control group, which were treated with norfloxacin, and their clinical symptoms worsened in severity starting on day 3, peaking on day 8 and recovering by day 15. Stool blood analysis showed cessation of bleeding by day 10, correlating with decreased colitis disease activity and an absence of diarrhoea or rectal bleeding (Fig. 2d). Furthermore, the colons of the vehicle-treated mice were significantly shorter, indicating the presence of inflammation (Fig. 3a). In contrast, the colons of the MESH-treated mice developed few symptoms and displayed normal lengths (Fig. 3b). Histological examination confirmed the loss of crypts, severe focal ulceration, and inflammation in the vehicle-treated mice (Fig. 3c), whereas the mice treated with MESH showed few abnormalities and had lower histological scores (Fig. 3d). These data from DSS-induced colitis indicated that, compared with the untreated and positive controls, MESH reduced mucosal barrier damage and prevented colonic inflammation.

### MESH affects inflammatory cytokine gene and protein expression

**Figure 4:**
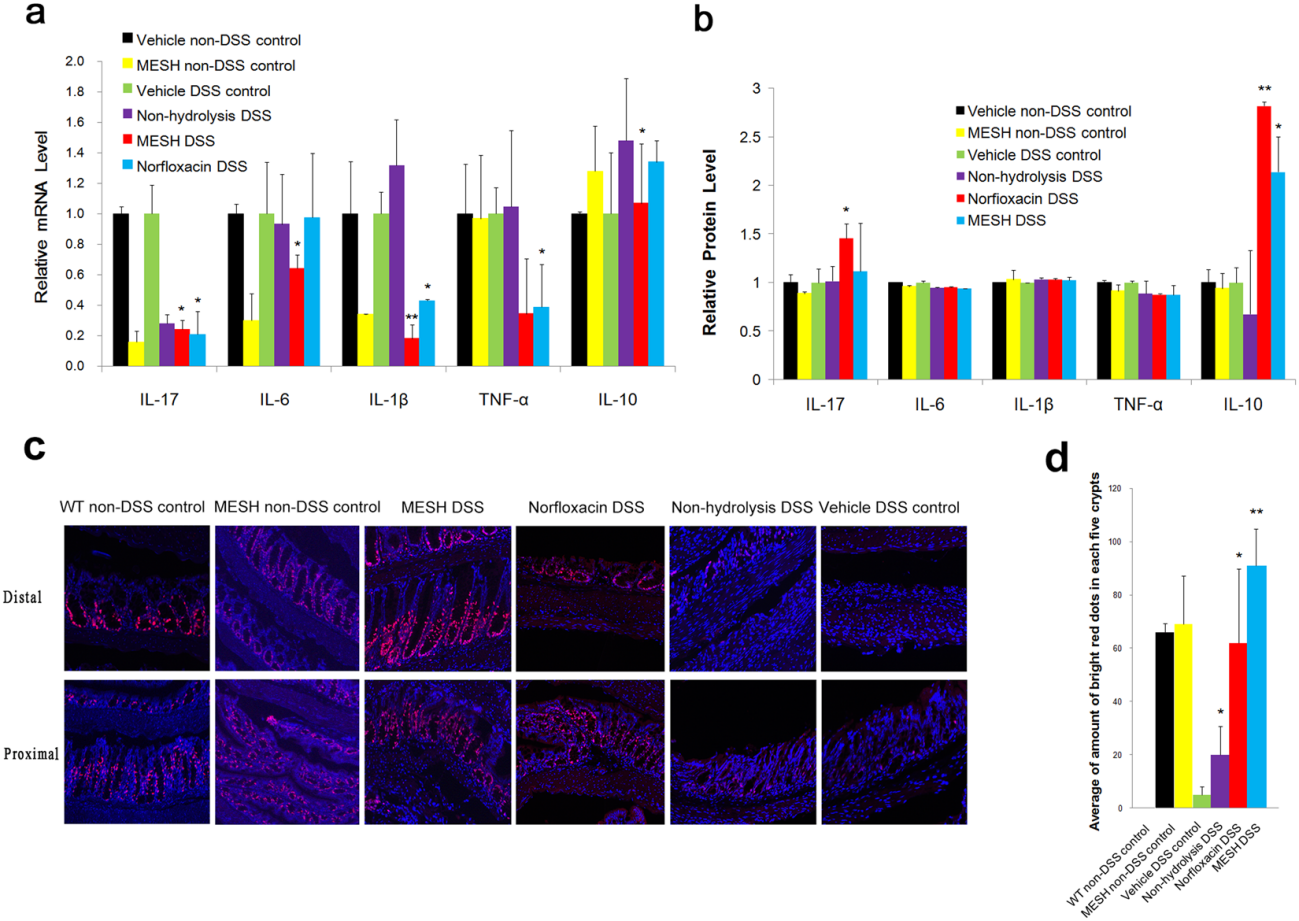
Colon cell proliferation and expression of inflammatory cytokine-encoding genes. (**a**) Relative mRNA levels of pro-inflammatory cytokines in the colons of mice. The gene expression of TNF-α, IL-6, IL-17 and IL-1β were decreased by MESH treatment. The gene expression of IL-10 showed no significance. ^*^P < 0.05; ^**^P < 0.01 versus the corresponding vehicle-DSS control. n = 5 in each group. (**b**) Relative protein levels of pro-inflammatory cytokines in the colons of mice. The protein expression ofIL-10 were increased by MESH treatment. There was only a slight decrease in IL-6 and TNF-α expression. *P < 0.05; **P < 0.01 versus the corresponding vehicle-DSS control. n = 5 in each group. (**c**) Use of Ki-67 (red) immunostaining to observe cell proliferation in the mucosa, with nuclei counterstained with DAPI (blue). The WT non-DSS control is displayed as a normal colon reference. Proximal and distal colons were analysed in each animal, and the average number of Ki-67+ cells per crypt per animal was determined between regions. MESH and norfloxacin treatments significantly elevated Ki-67+ cell numbers compared with the vehicle-DSS control, while MESH treatment stimulated greater numbers than those observed in the colons of control animals. Original magnification: 100×. (**d**) Quantification of Ki-67-positive cells per total crypt epithelial cells. ^*^P < 0.05; ^**^P < 0.01 versus the corresponding mouse group with DSS-vehicle treatment.

To assess whether MESH alters pro-inflammatory cytokine gene expression, we measured IL-6, IL-10, IL-1β, IL-17 and TNF-α mRNA levels, which are commonly used as indicators of inflammatory disease severity^18^. The sequences of the designed primers are shown in Table 1. The mice treated with MESH not only MESH-DSS group but also MESH non-DSS control group dramatically attenuated the increase in TNF-α, IL-17 and IL-1β expression levels resulting from DSS-induced colitis on day 10 (Fig. 4a) relative to those of the negtive and positive controls, although IL-6 expression was only slightly decreased and IL-10 expression slightly increased (Fig. 4a). These data support that the colons of the MESH-treated mice had decreased cellular inflammation compared with those of the control mice. Meanwhile, the IL-6, IL-10, IL-1β, IL-17 and TNF-α protein levels were measured by ELISA method. However, the results showed that only the protein levels of IL-10 expression increased significantly and that of TNF-α expression decreased slightly in the mice treated by MESH DSS-induced colitis on day 10 (Fig. 4b). There were no remarkable differences among the testing groups and controls to the IL-6, IL-1β, and IL-17. Therefore, MESH significantly increased the levels of IL-10 protein expression compared with the untreated control, which was consistent with the mRNA expression level results. MESH could decreased slightly TNF-α protein expression but significantly decreased TNF-α gene expression. These results suggested that MESH inhibited the acute inflammation by increasing both gene and protein expression of cytokine IL-10.

**Table 1:** Primers used for qPCR amplification.

Gene	Primer Nucleotide Sequences
TNF-α	Forward 5′-TCAGCCTCTTCTCATTCCTG-3′
	Reverse 5′-CAGGCTTGTCACTCGAATTT-3′
IL-1β	Forward 5′-CCAAAAGATGAAGGGGTGCTGCT-3′
	Reverse 5′-ACAGAGGATGGGCTCTTCTT-3′
IL-6	Forward 5′-ATAGTCCTTCCTACCCCAATTTCC-3′
	Reverse 5′-CTGACCACAGTGAGGAATGTCCAC-3′
IL-17	Forward 5′-TTTAACTCCCTTGGCGCAAAA-3′
	Reverse 5′-CTTTCCCTCCGCATTTGACAC-3′
IL-10	Forward 5′-CCACATGCTCCTAGAGCTGC-3′
	Reverse 5′-CCTTAAAGTCCTGCATTAAGGAGTCG-3′
GADPH	Forward 5′-GTCGTGGAGTCTACTGGT-3′
	Reverse 5′-TGCTGACAATCTTGAGTGAG-3

### MESH protects colon recovery from colitis

**Figure 5:**
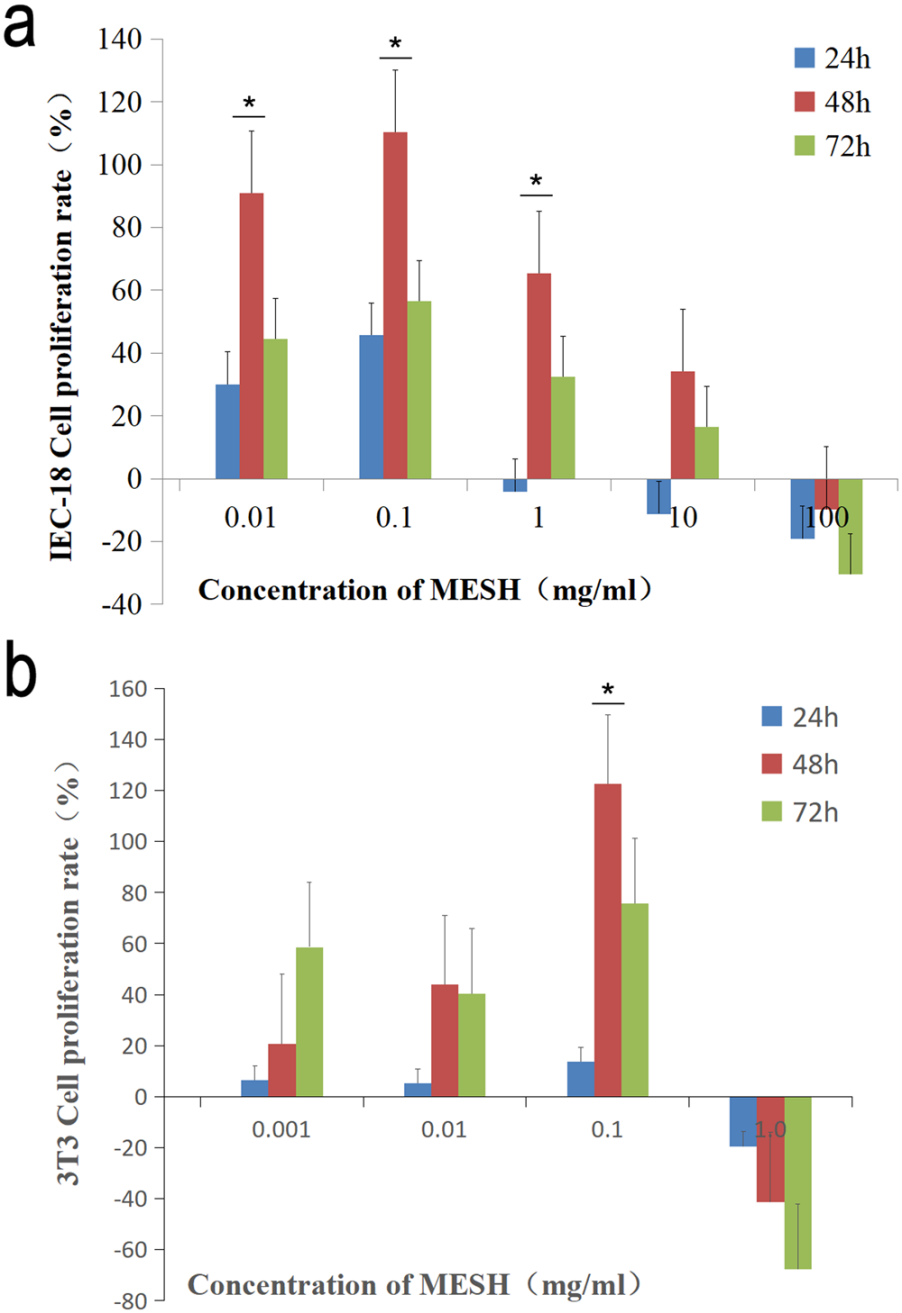
MESH promotes cell proliferation *in vitro*. (**a**) MESH promoted IEC-18 cell proliferation depending on the MESH concentration used. (**b**) MESH promoted IEC-18 cell proliferation, depending on the MESH concentration used. *P < 0.05 vs vehicle.

Intestinal stem cells proliferate at the base of intestinal crypts, supplying the epithelium with a continuous supply of differentiated cells with a 3–5 day turnover^19^. To explore the parameters of colonic barrier function with respect to MESH treatment, cell proliferation was investigated *in vitro* following MESH feeding. The number of Ki-67+ cells, a marker of proliferation, was determined following DSS-induced colitis^20^. Compared with the vehicle controls, MESH dramatically increased the number of Ki-67 markers in the colon (Fig. 4c) to levels greater than those observed in the animals without DSS treatment, whereas little affect was observed in the group provided non-hydrolysed meal (Fig. 4d). These results suggest that MESH promotes intestinal proliferation, possibly stimulating recovery following inflammation. To further verify this suggestion, IEC-18 and NIH 3T3 normal cells were respectively treated by MESH with different concentration of 0.001, 0.01, 0.1, 1, 10 and 100 mg/ml for 24, 48 and 72 hours (Fig. 5a and b). The results showed that MESH could promote not only 3T3 cells but also IEC-18 cells proliferation depended on the concentration. The optimal concentration is 0.1 mg/ml for 48 hours. Therefore, MESH could protect colon recovery from colitis.

## Discussion

In this study, we observed that hydrolysates derived from *Schizochytrium* sp. meal have potent anti-colitic activity in an experimental colitis mouse model. At least part of the anti-colitic mechanism of MESH is through the promotion of crypt cell proliferation through the upregulation of Ki-67, a key marker of cell proliferation, and this action results in the repair of the colonic mucosa. Additionally, MESH inhibited the expression of cytokines through the downregulation of TNF-α, IL-1β, IL-6 and IL-17 and slight upregulation of IL-10, thereby reducing colonic inflammation.

The microalgae *Schizochytrium* sp. is a food resource approved by the FDA (Food & Drug Administration, USA), SFDA (State Food & Drug Administration, China) and governments of other countries for bioengineering to producing DHA. Large amounts of DHA are produced around the world, but the resulting meal is typically discarded after the DHA extraction process. Studies have suggested that *Schizochytrium* sp. products can alleviate skin inflammation and improve wound healing in mice though a mechanism involving DHA^21^. In addition, oil fractions derived from *Schizochytrium* sp. have been reported to increase levels of the anti-inflammatory cytokine TGF-β^22^. However, whether the proteins in *Schizochytrium* sp. play a causative role in improving wound healing or inhibiting inflammation is unclear. In this study, we hydrolysed the meal produced following DHA extraction with enzymes and used the resulting hydrolysates to assess their anti-inflammation activities. These results showed that the hydrolysates had significant anti-inflammatory functions in murine models.

Since the hydrolysates exhibited an excellent anti-inflammatory effect, we wanted to examine their composition. Therefore, we further analysed the peptide profile resulting from the enzymatic treatment of the *Schizochytrium* sp. meal. The content of the hydrolysate fraction, named MESH, was identified by LC-MS/MS, which showed the presence of numerous peptides. The MWs of these peptides were almost 1 kDa or less, and it is commonly observed that the same several peptides may have the same MW. A bioinformatic approach was used to explore what types of proteins these peptides were derived from. We first searched the sequences of known proteins in *Schizochytrium* sp. in GenBank. Several proteins have been found in *Schizochytrium* sp., including polyunsaturated fatty acid synthase subunits A, B and C, elongation factor 1-alpha, actin, beta tubulin and type I fatty acid synthase^23,24^. The predicted cleavage sites for these proteins were generated for trypsin and pepsin, and this theoretical spectrum of MWs was then compared with the experimental spectrum (Supplementary Table 2) by AQS. We subsequently analysed the peptides and confirmed using UPLC/Q-TOF-MS that 32 had MWs that matched those of the theoretical peptide MWs, and only two MWs were unaccounted for. These 32 molecules corresponded to 172 peptide sequences, including those with anti-inflammatory capacities. Ten of the 32 peptides were present at high concentrations. All the experiments described above were performed to examine the components of MESH. Future studies are needed to synthesize these theoretical peptides and test their anti-inflammatory capacities with the goal of identifying the mechanisms through which these hydrolysed peptides function.

Many studies using the DSS-induced IBD model have focused on delaying and preventing inflammation. For example, Liu *et al*. reported onvitamin D receptor (VDR) inhibition of experimental colitis^25^, whereas MESH promoted recovery, demonstrating healing rather than simply preventing a worsening of symptoms. Our results showed that the clinical scores in the treatment and control groups were similar. The MESH treatment reversed the progression of DSS-induced colitis at an intermediate point and prevented acute inflammation, whereas the control DSS group continued to worsen with respect to clinical symptoms for an additional week. Our results are different from those observed for the inhibition of intestinal inflammation by VDR and suggest that MESH protected wound healing. Furthermore, histological examinations also confirmed that some degree of wound healing occurred after colitis. The colons of mice treated with MESH began to display a more normalized colonic epithelium, while those of the control group still lacked crypt formation and mucosal organization. Ki-67 is a nuclear indicator of cell proliferation to be abundant near the crypt base and diminishes during the normal differentiation and migration of epithelial cells to the colonic surface^26^.

In the current work, MESH enhanced Ki-67, indicating a role in growth and differentiation. In DSS-induced colitis, the roles of TNF-α and IL-1β differ in origin and function; TNF-α is more specific to colon epithelial cells, and IL-1β is more specific to myeloid cells^27^. Our studies showed that MESH lowered the expression of TNF-α but had no significant effect on IL-1β. These results suggest that the mechanism by which MESH promoted intestinal repair was partially related to an interaction with the epithelial cell compartment. MESH promoted IEC18 cell proliferation, as detected by MTT *in vitro* (Fig. 5a), suggesting that MESH could promote intestinal cell proliferation to facilitate would healing. TNF-α is a pro-inflammatory mediator associated with ulcerative colitis that is expressed at high levels in the colonic mucosa of patients with ulcerative colitis and has been the target of anti-TNF-α agents in human trials^28,29^. In our work, MESH had an inhibitory effect on the elevated TNF-α observed in the control animals. In addition to targeting intestinal inflammation, we observed that the hydrolysates derived from *Schizochytrium* sp.also mitigated inflammation at peripheral sites in response to local injurious stimuli. MESH also promoted the proliferation of normal mouse 3T3 cells (Fig. 5b), further evidence of how MESH is related to wound healing and inflammation reduction.

In conclusion, in this report, we revealed a new application of a by-product produced by bioengineered *Schizochytrium* sp. following DHA extraction, opening a new avenue of anti-inflammatory research. The use of *Schizochytrium* sp. has largely focused on the fact that it is an excellent source of DHA in industrial agriculture, and large amounts of biomass are available as starting material following DHA processing. We demonstrated that the hydrolysed product of *Schizochytrium* sp. can be used to mitigate inflammation in some common inflammatory diseases. This study suggests that the use of MESH may be a useful strategy for the treatment of inflammatory diseases.

## Methods

Male C57BL/6 mice were used to establish a bowel disease model of acute DSS-induced colitis. All experimental protocols and animal care were carried out in accordance to the guidelines approved by the Institution Animal Ethical Committee (IAEC) at the University of Fujian Medical University, Fuzhou, China.

### MESH preparation

The meal produced following DHA extraction from bioengineered *Schizochytrium* sp. was supplied by Runke Bioengineering Corporation of China. After dialysis, the meal was digested with pepsin, trypsin or both enzymes as previously described^15^. The hydrolysate obtained from the two-enzyme process was filtered and freeze dried into a powder (MESH). Briefly, the *Schizochytrium* sp. meal was ground into a powder and then was resuspended in PBS at a concentration of 10% w/v. The liquid was digested with pepsin (3% w/v) at the optimal pH value of 2 for 1 h at 37 °C and then by trypsin (3% w/v) under the optimal pH value of 7 for 5 h at 37 °C. Next, the temperature was increased to 60 °C for 10 min to inactivate the proteases. Next, the hydrolysates were centrifuged at 5000 × g and the supernatant was transferred into a large centrifuge tube. The hydrolysates were filtered through membrane filters (Millipore) with a MWCO of 1 kDa and centrifuged (5000 × g, 30 min) to separate the hydrolysate into two parts: one with an MWCO of greater than 1 kDa and one with an MWCO of less than 1 kDa. The part containing products with MWs of less than 1 kDa, named MESH, was then collected and dried at −40 °C using a freeze dryer (Mill-rock Technology) at 1 bar pressure for 24 h^30^. The resulting powders were used for the further experiments.

The MESH was analysed by UPLC/Q-TOF mass spectrometry (Waters, Massachusetts, USA). ESI-MS was performed by direct infusion of the aqueous extract in both the negative and positive modes. Mass spectra were acquired and scanned at a range between 300 and 1000 Da^31^, and the general conditions used were as follows: a source temperature of 80 °C and a desolvation temperature of 400 °C, a capillary voltage of 3.0 kV and a cone voltage of 50 V, and a desolvation gas flow of 800 L·h^−1^ were used.

The MESH was prepared at different concentrations between 1 and 100 mg·ml^−1^. The Non-hydrolysed *Schizochytrium* sp. meal, after desalting with the same concentration of the hydrolysates, was prepared as a control. After determining the correct dosage for the adult mice, the clinical drug norfloxacin, used at an optimal concentration of 5 mg·ml^−1^, was used as a positive control.

### DSS-induced colitis model assay

Male C57BL/6 mice were purchased from Slaccas, Shanghai, China. Mice (weighing 18–22 g) were studied using a DSS-induced colitis model. The mice were randomly assigned six groups of 7 mice per group and were administered PBS (0.9%) as a vehicle non-DSS control or vehicle-DSS control, norfloxacin (25 mg·kg^−1^) as a DSS-positive control, MESH (500 mg·kg^−1^) as an MESH-DSS or MESH-non-DSS control, and non-hydrolysed meal (500 mg·kg^−1^) as a DSS control (Supplementary Table 1). Each mouse was treated with 100 μL of the appropriate sample through intragastric administration once daily for 21 days.

Four groups of mice (MESH-DSS, norfloxacin-DSS, non-hydrolysed meal DSS and vehicle-DSS control) were provided 2.5% DSS (MP Biomedicals, Solon, Ohio, USA) dissolved in the drinking water for 1 cycle consisting of 5 days of DSS water and 16 days of tap water. The other control groups were not provided DSS. The body weights, stool consistency, and GI bleeding of all the mice were monitored daily. Clinical scores and colonic damage scores were estimated as detailed previously^32,33^. Colons were collected immediately after sacrifice, and the mucosa was scraped to isolate the total RNA. Colonic histology were performed using the ‘Swiss roll’ technique^34^, and histological scores were graded according to a previously published system^35^.

### Fecal occult blood measurements

Faecal occult blood detection was performed using the pyramidon semi-quantitative detection method. Aminopyrine was used as a colour indicator under acidic conditions with the presence of H_2_O_2_. It was assessed the reaction of stool blood with haemoglobin to produce purple-blue and purple-red colours. In the interpretation of the results, both positive and negative faecal occult blood were identified. The disease activity indexes (DAIs) were calculate by scoring weight loss, diarrhea and rectal bleeding based on the scoring system shown in Supplementary Table 3^36^. Curves of body weight loss and DAI were drawn to observe the changes in disease severity over the course of the experiment.

### Histology and immunohistochemical staining

Freshly dissected colons were split into two parts. One part was prepared using swiss rolls technique^37^ and was fixed overnight with 4% formaldehyde in PBS (pH 7.4), processed, and embedded in paraffin wax. Tissues were cut into 5-μm sections with a slicer (Leica microtome 2030). The colonic morphology was examined using haemotoxylin and eosin (H&E) staining. Histological tissues were observed and photographed using a Leica DMIRE2 microscope. The other part of the colon was scraped to isolate total RNA or proteins.

### Semi-quantitative RT-PCR

Total RNA from the colonic mucosa of mice was extracted using TRIzol reagent (Invitrogen, USA). First-strand cDNA templates were synthesized using AMV reverse transcriptase (Promega, USA)^38^ with hexanucleotide random primers. Conventional PCR was carried out in a BioRad thermocycler. Quantitative real-time PCR amplification was carried out using specific primers designed based on cDNA sequences deposited in the GenBank database^39^ (Table 1). Real-time PCR was performed in a Bio-rad CFX-96 Real-Time PCR system using SYBR green PCR reagent kits (Bio-rad). Briefly, each of the 40 cycles consisted of denaturation at 95 °C for 10 s, primer annealing at 55 °C for 20 s, and extension at 72 °C for 30 s after an initial hot start at 95 °C for 30 s, with a final incubation at 72 °C for 10 min. The relative amount of transcripts was calculated using the 2^−ΔΔCt^ formula as the described^40^, where DCt is the value from the threshold cycle (Ct) value of the treated sample subtracting the Ct value of the untreated or zero time-point control sample. The relative amount of the sample mRNA was normalized to the GAPDH mRNA.

### Sandwich ELISA assay

The protein expression of TNF-α, IL-6, IL-17, IL-10 and IL-1β were determined using a mouse-specific ELISA kit (Abbkine, Inc., Wuhan, China). The protein lysate was taken from colonic mucosa. The procedure was according to the manusfacturer’s instructions. Briefly, an antibody specific for Cytokines (IL-6, IL-10, IL-1β, IL-17 and TNF-α) had been pre-coated onto a microplate. One hundred microliter of diluted total protein were respectively added into the wells in a 96-well plate and incubated at 37 °C for 1 hour and then washed three times with 200 μl of wash solution. With 100 μl of a biotin-conjugated antibody specific for cytokines were added and incubated for 1 hour at 37 °C and then washed three times with 200 μl of wash solution. It was incubated with 100 μl of the working dilution of Streptavidin HRP for 30 min at 37 °C. Subsequently, 100 μl of HRP substrate was added and incubated for 15 min at 37 °C. The enzyme reaction was stopped by adding 50 μl of stop solution in dark. It was determined at 450 nm of absorbance and was corrected at 570 nm. The protein levels of the IL-6, IL-10, IL-1β, IL-17 and TNF-α were detected by this ELISA method.

### Ki-67 immunohistochemical analysis

Sections (5 μm) were cut from the prepared ‘Swiss roll’ colonic tissue samples. Slides were blocked for 1 hour at room temperature and incubated overnight at 4 °C with a (1:100) primary rat anti-mouse Ki-67 monoclonal antibody (TEC-3; Dako, Copenhagen, Denmark). After washing with PBS, the sections were incubated for 30 minutes with a biotinylated anti-rat IgG antibody (1:100 in 1% rabbit serum) for 1 hour at room temperature, after which they were rinsed and reacted with fluorescein isothiocyanate–conjugated streptavidin (Vector Laboratories, Burlingame, CA) for 60 minutes. DAPI was used for nuclear counterstaining. To assess the cell proliferation activity, the number of Ki-67-positive cells per total crypt epithelial cells was enumerated. The Ki-67 labelling index was defined as the percentage of Ki-67-positive cells per crypt^41^. All the sections were blinded and analysed by two investigators.

### Cell culture

IEC18 cells originating from rat ileum epithelium were obtained from The University of Chicago (Chicago, IL, USA). The maintenance medium used was Dulbecco’s modified Eagle’s medium (DMEM) with high glucose (4.5 g·l^−1^), 4 mM L-glutamine, and 110 mg·L^−1^ sodium pyruvate, supplemented with 5% foetal bovine serum (Gibco^®^ Cell Culture, Melbourne, VIC, Australia) and 0.1 U·ml^−1^ bovine insulin (Sigma, St. Louis, MO, USA). Cells were cultured at 37 °C with a 5% CO_2_ atmosphere at 90% relative humidity. Six concentrations of MESH were prepared (0.001, 0.01, 0.1, 1, 10, and 100 mg/ml) and added to the cell culture plates for 24, 48 or 72 h.

The mouse fibroblast BALB/c 3T3 cell line was obtained from the Applied Biotechnology Institute(Fuzhou, FJ, China). In each well of a 96-well plate, 5 × 10^4^ cells were seeded and cultured in DMEM supplemented with 10% foetal bovine serum and incubated at 37 °C with a 5% CO_2_ atmosphere for 24, 48 or 72 h.

MESH was prepared at five concentrations (0.001, 0.01, 0.1, 1, 10 and 100 mg·ml^−1^) and added to different wells of the cell culture plates.

### MTT assay

IEC-18 or 3T3 cells were respectively seeded into 96-well plates with 5 × 10^4^ cells per well and then put them into a CO_2_ incubator for 24 hours. The cells were treated by MESH with concentration of 0.01, 0.1, 1, 10 and 100 mg/ml for 24, 48 and 72 hours, respectively. After the cells were washed with PBS, 50 µl of MTT solution from the Stock (5 mg/ml) was added into each well and then incubated in CO_2_ incubator in the dark for 4 hours. The MTT solution was removed and 150 μl of DMSO were added into each well for 15 min at room temperature. Mixed each sample using a pipette and read absorbance at 570 nm.

### Statistical analysis

Data values were presented as means ± SD. Statistical comparisons were carried out using unpaired two-tailed Student’s *t*-test for two group comparisons, and for three or more group comparisons two-way analysis of variance (ANOVA) with a Student-Newman-Keuls post-hoc test was used. P < 0.05 were considered statistically significant.

## Electronic supplementary material

Dataset 1
