# Supplementary material for: Marine microalgae bioengineered Schizochytrium sp. meal hydrolysates inhibits acute inflammation
Source: Sci Rep. 2018 Jun 29;8:9848. doi: 10.1038/s41598-018-28064-y (PMC6026148; doi:10.1038/s41598-018-28064-y)
Supplement: Supplementary file 1 — Dataset 1 [file 41598_2018_28064_MOESM1_ESM.docx]

**Marine microalgae bioengineered *Schizochytrium* *sp*. meal hydrolysates inhibits acute inflammation**

Xiaoli Wang^1^, Heng Wang^1^, Joseph F Pierre^2^, Sheng Wang^3^, Huifang Huang^4^, Jun Zhang^1^, Shuangzhen Liang^1^, Qingzhu Zeng^1^, Chenqing Zhang^1^, Meijuan Huang^4^, Chengxu Ruan^1^, Juan Lin^5^, Hao Li^1,2,6*^

^1^Institute of Applied Biotechnology, College of Biological Science and Technology, Fuzhou University, Fuzhou, Fujian 350108, China. ^2^Section of Gastroenterology, Hepatology, and Nutrition, Department of Medicine, The University of Chicago, Chicago, IL 60637, USA. ^3^Department of Human Genetics, The University of Chicago, Chicago, IL 60637, USA. ^4^Central Laboratory, Fujian Medical University Union Hospital , Fuzhou, 350001, China..^5^Fujian Key Laboratory of Marine Enzyme Engineering, Fuzhou University.^6^Fujian LandhowbioTech. Corp.,Ltd., Fuzhou, Fujian 350108, China. ^§^ Authors equally contribute to this work. *Correspondence and requests for materials should be addressed to Hao Li ([haoli@fzu.edu.cn](mailto:haoli@fzu.edu.cn))

**Supplementary information as follows,**

Supplementary table 1. The xylene induced mouse auricle inflammation administration table

| Group | Gavage | Dose C/mg·kg^-1^ |
| --- | --- | --- |
| Vehicle | PBS | - |
| Non-hydrolysis | Non-hydrolysis | 500 |
| Dexamethasone | Dexamethasone | 10 |
| Lower concentration | MESH | 250 |
| Middle concentration | MESH | 500 |
| High concentration | MESH | 1000 |

Supplementary table 2. Theory peptide sequences derived from *Schizochytrium sp.*were found in the experimental test spectrum

| MESH  MW | Theory peptides MW | No | Theory peptides sequence | Protein name |
| --- | --- | --- | --- | --- |
| 313.1548 | 313.34 | 1 | HSA | type I fatty acid synthase |
| 318.1812 | 318.29 | 2 | DNA | type I fatty acid synthase |
|  | 318.30 | 3 | NGE | elongation factor 1-alpha |
|  | 318.32 | 4 | DGQ | elongation factor 1-alpha |
|  | 318.36 | 5 | GSR | polyunsaturated fatty acid synthase subunit B/C, type I fatty acid synthase |
|  | 318.36 | 6 | SGR | actin |
|  | 318.36 | 7 | DGK | polyunsaturated fatty acid synthase subunit A/C |
| 332.2162 | 332.36 | 8 | SNL | type I fatty acid synthase |
|  | 332.38 | 9 | ASR | polyunsaturated fatty acid synthase subunit B/C |
|  | 332.38 | 10 | DAK | beta tubulin,type I fatty acid synthase |
|  | 332.38 | 11 | SAR | polyunsaturated fatty acid synthase subunitB/C |
|  | 332.39 | 12 | VSQ | polyunsaturated fatty acid synthase subunit A/C, acyl carrier protein |
|  | 332.39 | 13 | EGK | polyunsaturated fatty acid synthase subunit B/C |
|  | 332.39 | 14 | GEK | polyunsaturated fatty acid synthase subunitB/C, type I fatty acid synthase |
| 344.2540 | 344.40 | 15 | TPQ | polyunsaturated fatty acid synthase subunit C, type I fatty acid synthase |
|  | 344.42 | 16 | VNL | beta tubulin,polyunsaturated fatty acid synthase subunit C |
|  | 344.44 | 17 | AVR | polyunsaturated fatty acid synthase subunit A |
|  | 344.44 | 18 | TPK | polyunsaturated fatty acid synthase subunit A/C, elongation factor 1-alpha |
|  | 344.44 | 19 | VAR | type I fatty acid synthase |
|  | 344.44 | 20 | AAAL | type I fatty acid synthase |
|  | 344.45 | 21 | VVQ | type I fatty acid synthase |
|  | 344.46 | 22 | GVVA | polyunsaturated fatty acid synthase subunit C |
|  | 344.49 | 23 | VVK | type I fatty acid synthase,acyl carrier protein |
| 358.2697 | 358.36 | 24 | NPE | polyunsaturated fatty acid synthase subunit C, type I fatty acid synthase |
|  | 358.36 | 25 | PNE | type I fatty acid synthase |
|  | 358.42 | 26 | GPW | polyunsaturated fatty acid synthase subunit A/C |
|  | 358.42 | 27 | PSR | polyunsaturated fatty acid synthase subunit B/C |
|  | 358.42 | 28 | SPR | type I fatty acid synthase |
|  | 358.42 | 29 | DPK | polyunsaturated fatty acid synthase subunit C polyunsaturated fatty acid synthase subunit B |
|  | 358.44 | 30 | INL | polyunsaturated fatty acid synthase subunit C |
|  | 358.46 | 31 | AIR | type I fatty acid synthase |
|  | 358.46 | 32 | IAR | type I fatty acid synthase |
|  | 358.47 | 33 | IVQ | polyunsaturated fatty acid synthase subunit A/C |
|  | 358.47 | 34 | QVL | polyunsaturated fatty acid synthase subunit B/C |
|  | 358.48 | 35 | IGVA | polyunsaturated fatty acid synthase subunit A/C |
|  | 358.48 | 36 | VGIA | type I fatty acid synthase |
| 360.2132 | 360.38 | 37 | NVE | polyunsaturated fatty acid synthase subunit A/C |
|  | 360.39 | 38 | DAR | polyunsaturated fatty acid synthase subunit A |
|  | 360.40 | 39 | SSPA | polyunsaturated fatty acid synthase subunit C |
|  | 360.41 | 40 | VDGA | acyl carrier protein |
|  | 360.44 | 41 | DVK | polyunsaturated fatty acid synthase subunit A/C |
|  | 360.44 | 42 | ITQ | polyunsaturated fatty acid synthase subunit C |
|  | 360.44 | 43 | SVR | type I fatty acid synthase |
|  | 360.44 | 44 | VDK | polyunsaturated fatty acid synthase subunit C |
|  | 360.44 | 45 | VSR | polyunsaturated fatty acid synthase subunit B/C |
|  | 360.44 | 46 | SAAL | type I fatty acid synthase |
|  | 360.48 | 47 | TLK | beta tubulin |
| 374.2301 | 374.40 | 48 | ENL | polyunsaturated fatty acid synthase subunit A, type I fatty acid synthase |
|  | 374.42 | 49 | AER | polyunsaturated fatty acid synthase subunit B/C |
|  | 374.43 | 50 | GDAL | polyunsaturated fatty acid synthase subunit C |
|  | 374.43 | 51 | GDIA | polyunsaturated fatty acid synthase subunit C |
|  | 374.43 | 52 | GIDA | polyunsaturated fatty acid synthase subunit A/C |
|  | 374.43 | 53 | PSTA | type I fatty acid synthase |
|  | 374.46 | 54 | DIK | actin,elongation factor 1-alpha |
|  | 374.46 | 55 | LDK | polyunsaturated fatty acid synthase subunit B |
|  | 374.47 | 56 | TVR | type I fatty acid synthase |
|  | 374.47 | 57 | VEK | polyunsaturated fatty acid synthase subunit B/C |
|  | 374.47 | 58 | VTR | polyunsaturated fatty acid synthase subunit C |
|  | 374.48 | 59 | GVSL | type I fatty acid synthase |
|  | 374.48 | 60 | SGVL | polyunsaturated fatty acid synthase subunit A/C, type I fatty acid synthase |
|  | 374.48 | 61 | VSGL | putative superoxide dismutase,elongation factor 1-alpha |
| 378.2375 | 378.42 | 62 | TSTA | type I fatty acid synthase |
|  | 378.45 | 63 | SVCA | acyl carrier protein |
|  | 378.48 | 64 | MTQ | actin |
|  | 378.48 | 65 | TMQ | polyunsaturated fatty acid synthase subunit A/C |
|  | 378.49 | 66 | TMGA | polyunsaturated fatty acid synthase subunit C |
|  | 378.52 | 67 | MTK | polyunsaturated fatty acid synthase subunit C, type I fatty acid synthase |
| 392.2534 | 392.44 | 68 | SSSL | actin |
|  | 392.45 | 69 | TTTA | actin |
|  | 392.50 | 70 | DMK | type I fatty acid synthase |
|  | 392.50 | 71 | MSR | type I fatty acid synthase |
|  | 392.53 | 72 | VFK | polyunsaturated fatty acid synthase subunit B |
| 415.2905 | 415.49 | 73 | PDGK | type I fatty acid synthase |
|  | 415.49 | 74 | PSGR | polyunsaturated fatty acid synthase subunit A/C |
|  | 415.51 | 75 | AVNL | beta tubulin |
|  | 415.51 | 76 | GINL | type I fatty acid synthase |
|  | 415.51 | 77 | INGL | polyunsaturated fatty acid synthase subunit C |
|  | 415.52 | 78 | IQR | polyunsaturated fatty acid synthase subunit B/C, type I fatty acid synthase |
|  | 415.52 | 79 | LQR | polyunsaturated fatty acid synthase subunit B |
|  | 415.53 | 80 | LGAR | polyunsaturated fatty acid synthase subunit B |
|  | 415.55 | 81 | VGIGA | polyunsaturated fatty acid synthase subunit A/C |
| 417.2392 | 417.43 | 82 | ENR | polyunsaturated fatty acid synthase subunit B/C |
|  | 417.45 | 83 | QDR | type I fatty acid synthase |
|  | 417.47 | 84 | GSSPA | polyunsaturated fatty acid synthase subunit A/C |
|  | 417.47 | 85 | NVW | polyunsaturated fatty acid synthase subunit A/C, type I fatty acid synthase |
|  | 417.51 | 86 | SAAGL | type I fatty acid synthase |
|  | 417.51 | 87 | SGVR | acyl carrier protein |
|  | 417.54 | 88 | ISAK | polyunsaturated fatty acid synthase subunit A |
|  | 417.55 | 89 | AVTK | polyunsaturated fatty acid synthase subunit A |
| 431.2606 | 431.47 | 90 | GINE | type I fatty acid synthase |
|  | 431.47 | 91 | GNIE | putative superoxide dismutase,elongation factor 1-alpha |
|  | 431.49 | 92 | NWL | type I fatty acid synthase |
|  | 431.53 | 93 | TPSK | polyunsaturated fatty acid synthase subunit A/C |
|  | 431.54 | 94 | VGEK | polyunsaturated fatty acid synthase subunit C |
|  | 431.55 | 95 | GVVSA | type I fatty acid synthase |
| 445.2669 | 445.49 | 96 | NAEL | type I fatty acid synthase |
|  | 445.51 | 97 | DPSK | polyunsaturated fatty acid synthase subunit C |
|  | 445.51 | 98 | EAAR | type I fatty acid synthase |
|  | 445.51 | 99 | PSDK | elongation factor 1-alpha |
|  | 445.52 | 100 | QGEL | polyunsaturated fatty acid synthase subunit B/C |
|  | 445.54 | 101 | QWL | type I fatty acid synthase |
|  | 445.54 | 102 | VNTL | type I fatty acid synthase |
|  | 445.55 | 103 | DIAK | polyunsaturated fatty acid synthase subunit B/C |
|  | 445.56 | 104 | VATR | polyunsaturated fatty acid synthase subunit B/C |
| 473.2945 | 473.54 | 105 | INDL | acyl carrier protein |
|  | 473.54 | 106 | NIDL | polyunsaturated fatty acid synthase subunit C |
|  | 473.57 | 107 | IDVQ | polyunsaturated fatty acid synthase subunit A/C |
|  | 473.58 | 108 | GVDIA | polyunsaturated fatty acid synthase subunit A/C |
|  | 473.61 | 109 | QTIL | polyunsaturated fatty acid synthase subunit B/C |
|  | 473.61 | 110 | DIVK | type I fatty acid synthase |
|  | 473.61 | 111 | VDIK | type I fatty acid synthase |
|  | 473.61 | 112 | VIDK | polyunsaturated fatty acid synthase subunit C |
|  | 473.63 | 113 | VGSVL | polyunsaturated fatty acid synthase subunit A/C |
|  | 473.63 | 114 | VVSGL | type I fatty acid synthase |
| 489.2584 | 489.49 | 115 | GNNW | beta tubulin |
|  | 489.50 | 116 | DNIE | type I fatty acid synthase |
|  | 489.53 | 117 | NGGDK | type I fatty acid synthase |
|  | 489.57 | 118 | SEAAL | type I fatty acid synthase |
|  | 489.61 | 119 | TTLR | polyunsaturated fatty acid synthase subunit A |
|  | 489.67 | 120 | MALR | polyunsaturated fatty acid synthase subunit C |
| 493.2674 | 493.65 | 121 | PIHK | type I fatty acid synthase |
| 516.2991 | 516.59 | 122 | GNVDL | polyunsaturated fatty acid synthase subunit B/C |
|  | 516.61 | 123 | TGPSR | type I fatty acid synthase |
|  | 516.63 | 124 | AVNTL | type I fatty acid synthase |
|  | 516.67 | 125 | AWIK | type I fatty acid synthase, polyunsaturated fatty acid synthase subunit B |
|  | 516.70 | 126 | TGVIK | elongation factor 1-alpha |
|  | 516.70 | 127 | VVTAK | type I fatty acid synthase |
| 530.3167 | 530.57 | 128 | YDSF | polyunsaturated fatty acid synthase subunit B/C |
|  | 530.63 | 129 | AAVDR | polyunsaturated fatty acid synthase subunit A |
|  | 530.63 | 130 | PEASK | polyunsaturated fatty acid synthase subunit B/C |
|  | 530.63 | 131 | SPQSL | type I fatty acid synthase |
|  | 530.63 | 132 | GANAAK | type I fatty acid synthase |
|  | 530.67 | 133 | AALEK | polyunsaturated fatty acid synthase subunit C |
|  | 530.67 | 134 | EAAIK | polyunsaturated fatty acid synthase subunit A |
|  | 530.72 | 135 | TGIIK | elongation factor 1-alpha |
| 544.3008 | 544.63 | 136 | VPGSDA | polyunsaturated fatty acid synthase subunit A/C |
|  | 544.70 | 137 | AVIDK | polyunsaturated fatty acid synthase subunit C |
|  | 544.70 | 138 | PCVVQ | polyunsaturated fatty acid synthase subunit C |
|  | 544.71 | 139 | VSQVL | polyunsaturated fatty acid synthase subunit A |
|  | 544.71 | 140 | VAVEK | polyunsaturated fatty acid synthase subunit A |
| 546.3067 | 546.64 | 141 | NGMPE | polyunsaturated fatty acid synthase subunit C |
|  | 546.68 | 142 | DVSVK | polyunsaturated fatty acid synthase subunit A/C |
|  | 546.69 | 143 | HMPY | polyunsaturated fatty acid synthase subunit C |
|  | 546.70 | 144 | PGVGAF | type I fatty acid synthase |
| 567.4307 | 567.76 | 145 | MTAVF | type I fatty acid synthase |
| 576.2972 | 576.73 | 146 | CVIGW | type I fatty acid synthase |
|  | 576.77 | 147 | VMTAR | type I fatty acid synthase |
| 589.3315 | 589.61 | 148 | DGVNDA | type I fatty acid synthase |
|  | 589.69 | 149 | TIEDL | type I fatty acid synthase |
|  | 589.71 | 150 | TAQASL | type I fatty acid synthase |
|  | 589.75 | 151 | VPEVF | polyunsaturated fatty acid synthase subunit C |
|  | 589.76 | 152 | TPEML | type I fatty acid synthase |
|  | 589.81 | 153 | MQAIK | polyunsaturated fatty acid synthase subunit A |
| 628.3656 | 628.74 | 154 | AAEEPL | type I fatty acid synthase |
|  | 628.74 | 155 | IPDDVA | acyl carrier protein |
| 657.3654 | 657.83 | 156 | VPISGW | elongation factor 1-alpha |
| 674.3325 | 674.79 | 157 | HHDGPL | type I fatty acid synthase |
|  | 674.80 | 158 | SVSNIR | polyunsaturated fatty acid synthase subunit C |
|  | 674.81 | 159 | SEIVDL | type I fatty acid synthase |
| 701.3952 | 701.83 | 160 | AEIQNK | elongation factor 1-alpha |
|  | 701.83 | 161 | LDDAIR | polyunsaturated fatty acid synthase subunit B |
|  | 701.84 | 162 | QSAGINL | type I fatty acid synthase |
| 708.3329 | 708.82 | 163 | LGSNYR | polyunsaturated fatty acid synthase subunit B |
|  | 708.86 | 164 | NTAYIK | polyunsaturated fatty acid synthase subunit A |
| 719.3622 | 719.79 | 165 | VVDDGSE | polyunsaturated fatty acid synthase subunit A/C |
|  | 719.81 | 166 | DISSIDA | type I fatty acid synthase |
|  | 719.84 | 167 | CDVDIR | actin |
|  | 719.95 | 168 | IVVDFK | polyunsaturated fatty acid synthase subunit C |
| 805.3787 | 805.00 | 169 | TVITGSVE | polyunsaturated fatty acid synthase subunit A/C |
|  | 805.01 | 170 | DSVMPGSL | polyunsaturated fatty acid synthase subunit C |
|  | 805.93 | 171 | TPGDNVGF | elongation factor 1-alpha |
| 829.4411 | 829.04 | 172 | ILEETPK | elongation factor 1-alpha |
| 847.4106 | 847.08 | 173 | PTDISIMA | type I fatty acid synthase |

Supplementary table 3. Criteria for scoring disease activity index

| Score | Weight loss (%) | Stool consistency | Occult blood or gross bleeding |
| --- | --- | --- | --- |
| 0  1  2  3  4 | None  1-5  5-10  10-15  > 15 | Normal  Loose stool  Loose stool  Diarrhea  Diarrhea | Negative  Negative  Hemoccult positive  Hemoccult positive  Gross bleeding |
